# Rapid weight loss and combat athletes: a study on psychological resilience and mechanical hyperalgesia

**DOI:** 10.3389/fpsyg.2025.1545048

**Published:** 2025-01-22

**Authors:** Esin Çağla Çağlar, Levent Ceylan, Sermin Ağralı Ermiş, Furkan Çamiçi, Murat Eliöz, Recep Nur Uzun, Güner Çiçek, Muhammet Kusan, Mustafa Tolga Bayraktar, Fatma Neşe Şahin, Hamza Küçük

**Affiliations:** ^1^Faculty of Sport Sciences, Hitit University, Corum, Türkiye; ^2^Faculty of Sport Sciences, Ankara University, Ankara, Turkey; ^3^Yasar Doğu Faculty of Sport Sciences, Ondokuz Mayis Unersity, Samsun, Türkiye

**Keywords:** mechanical hyperalgesia, rapid weight loss, psychological resilience, combat athletes, kickboxers

## Abstract

**Introduction:**

The study investigates the effects of Rapid Weight Loss (RWL) on the levels of psychological resilience and mechanical pain sensitivity in elite kickboxers. The primary aim was to examine changes in psychological resilience subdimensions and pressure pain threshold (PPT) values in the thoracolumbar region before and after a one-month RWL period leading up to competition. Hypotheses included expectations of significant reductions in PPT values due to biomechanical and physiological changes during RWL, along with improvements in psychological resilience subdimensions due to structured routines and social support.

**Methods:**

Thirty-seven elite male athletes (age: 22.94 ± 1.98) participated in the study. Psychological resilience was assessed using the Psychological Resilience Scale for Adults, and mechanical pain sensitivity was evaluated through Pressure Pain Threshold (PPT) measurements. Measurements were conducted before and after the RWL period, spanning one month prior to competition.

**Results:**

The results revealed significant PPT values across all thoracolumbar segments after RWL (*p* < 0.001). Cohen’s *d* values indicated large effect sizes for these changes (*d* = 2.10–2.36). The L5 segment exhibited the largest PPT decrease (*t* = −10.45, *d* = 2.36), while the Th1 segment showed the smallest decrease (*t* = −8.23, *d* = 2.10). Measurements taken at 4 cm from the spine midline also demonstrated significant PPT reductions (*p* < 0.001), with the highest change recorded in the L5 segment (*t* = −9.78, *d* = 2.30). Psychological resilience subdimensions, including “structured style,” “social competence,” “future orientation,” and “social resources,” improved significantly after RWL (*p* < 0.05), while “family cohesion” and “personal strength” did not show significant changes. Athletes who previously ranked in tournaments exhibited higher psychological resilience, potentially due to enhanced self-confidence.

**Discussion:**

These findings align with literature suggesting that competitive success and optimism play crucial roles in resilience development. Personality traits and perceived social support further contributed to the observed resilience levels. This study highlights the multidimensional impact of RWL, emphasizing its detrimental effects on pain sensitivity and its mixed outcomes on psychological resilience. RWL-associated increases in mechanical hyperalgesia, especially in the lumbar region, were attributed to biomechanical and neurophysiological factors. Enhanced psychological resilience observed in certain subdimensions underscores the importance of structured and social support systems in mitigating RWL-induced stress. Future research should explore interventions to optimize resilience and manage pain during RWL periods, focusing on individualized support strategies for athletes. These findings contribute to understanding the interplay between psychological and physiological factors during RWL, practical insights for athletic training.

## 1 Introduction

Combat sports (CS) are characterized by different weight divisions, where athletes frequently compete in a lower weight category than their natural body mass to gain a competitive edge (Brechney et al., 2022). This strategy relies on RWL immediately before the official weigh-in, which is then followed by a period of rehydration and carbohydrate intake (Martínez-Aranda et al., 2023). This weight-cycling practice allows athletes to compete at a higher weight after weigh-ins, potentially offering an advantage over lighter opponents. However, the widespread use of RWL in CS often leads athletes to adopt extreme weight-loss methods that may compromise performance and health. Methods such as drastic caloric restriction, prolonged fasting, sauna use, and even the use of prohibited pharmaceutical aids are common, often leading to severe dehydration, impaired performance, and possible health risks (Lakicevic et al., 2020; Uddin et al., 2022). In addition, hormonal changes may also negatively affect physical performance (Küçük and Ceylan, 2022; Kucuk et al., 2024; Ünver et al., 2024).

In addition to performance-related drawbacks, RWL can negatively impact cognitive and physiological functions (Drid et al., 2019). Studies have highlighted the detrimental effects of these methods on cognitive aspects such as short-term memory, mood, and decision-making processes. For example, while rehydration may help return some cognitive measures to baseline, the transient decline in memory, focus, and anticipation skills due to RWL poses a significant risk, particularly in high-stakes, rapid-response environments like combat sports (Drid et al., 2024). Furthermore, RWL and repeated cycles of weight loss and regain—commonly known as “weight cycling”—have demonstrated adverse effects on physical health, particularly on the endocrine and renal systems. Symptoms associated with repeated RWL include hormonal imbalances and renal strain, which not only undermine the physical capabilities required for competition but also may have long-term health implications (Horswill, 1992; Uddin et al., 2022).

An often-overlooked impact of RWL is its effect on psychological resilience, particularly under competitive stress. Combat sports are inherently challenging, and athletes frequently experience heightened levels of competitive anxiety (CA), marked by cognitive (e.g., performance-related worry) and somatic (e.g., muscle tremors) symptoms (Rowland and Van Lankveld, 2019). Although moderate levels of anxiety may enhance performance for some, elevated CA generally correlates with performance declines due to cognitive interference and physiological stress responses. Anxiety symptoms like disrupted motor coordination and cortisol spikes exacerbate the physical and mental toll of CS, especially under RWL. Another factor that physically affects athletes during the RWL period is muscle soreness, which is perceived as a dull or aching sensation (Hanada et al., 2022). According to a study, chronic musculoskeletal pain is more common in the lower back compared to extremities such as elbows, hands, hips, knees and feet (Nakamura et al., 2011). From a histological point of view, the erector spinae muscle in the lower back contains more type I (slow) fibers than the vastus lateralis muscle (limb muscle) in humans (Staron et al., 2000; Mannion et al., 1997; Hanada et al., 2022). Functionally, lumbar muscles are involved in postural control rather than movement. It is not known whether these differences in fiber type composition and functional roles are related to the high prevalence of chronic pain in the lumbar muscles. Pressure pain threshold (PPT) may be a good measure of the magnitude of clinical muscle pain such as low back pain (Graven-Nielsen and Arendt-Nielsen, 2010; Hanada et al., 2022) because the pressure pain threshold (PPT) can be a good measure of the magnitude of clinical muscle pain, such as low back pain, because it is a simple, reliable and reproducible method; it can objectively assess the level of muscle tenderness and pain perception (Graven-Nielsen and Arendt-Nielsen, 2010; Mizumura et al., 2010). In a study by Abboud et al. (2019), pressure pain thresholds (PPTs) were assessed at four lumbar regions, specifically at the L2 and L4 segments located approximately 2.5 cm lateral to the spinous processes. The findings indicated that trunk flexion-extension exercises led to increased pain sensitivity (DOMS) and decreased functional capacity in the paraspinal muscles.

Therefore, PPT measurements can be a valuable tool not only to determine the severity of low back pain but also to assess changes in pain management and musculoskeletal functional capacity. The observed increases in muscle tenderness and decreases in functional capacity of paraspinal muscles, especially after exercise and rapid weight loss, increase the usefulness of PPT measurements in both clinical and rehabilitation processes. Future studies may support the use of PPT as part of individualized treatment plans by examining how pain sensitivity in the lumbar muscles changes with different exercise protocols. Finally, while strategies like RWL in combat sports may offer short-term physical advantages, the potential psychological and physiological costs are notable. The reduction of essential nutrients and dehydration not only threatens endurance and strength but can also lead to mood disturbances and weakened mental resilience. In a sport demanding rapid physical and mental adaptability, understanding the cognitive effects of RWL is crucial to better support athletes’ well-being and performance under competitive pressure.

This study aimed to examine the levels of psychological resilience and pressure pain thresholds (PPT) in elite kickboxers during the RWL period, focusing on changes in resilience subdimensions and PPT values. PPT was used as an objective measure of muscle tenderness, while resilience subdimensions provided insights into athletes’ adaptive capacity under competitive stress. The findings confirm the hypothesis that PPT values would significantly decrease across thoracolumbar segments, particularly in the lumbar region, due to increased biomechanical loading and dehydration. As expected, resilience subdimensions such as “structured style” and “social competence” improved after RWL, highlighting the role of structured routines and social support in enhancing psychological adaptation. Conversely, subdimensions like “family cohesion” and “personal strength” remained unchanged, suggesting variability in resilience responses.

The one-month follow-up during the RWL period likely limited the ability to capture long-term effects, as hypothesized. Future longitudinal studies are necessary to assess whether observed changes persist or evolve over time. By aligning the research goals with measurable outcomes, this study contributes practical insights into the physiological and psychological dimensions of RWL, providing actionable strategies to optimize athlete management during critical periods.

## 2 Materials and methods

### 2.1 Participants

Participants filled out informed consent forms. A total of 37 elite male kickboxers (age: 22.94 ± 1.98) participated in the study.

Inclusion criteria for participants:

- Male elite kickboxers aged between 18 and 25 years to ensure physiological and psychological homogeneity, as hormonal fluctuations and resilience levels can vary significantly outside this age range.
- Athletes with at least three years of professional experience in combat sports to focus on individuals accustomed to the physical and psychological demands of the sport.
- Participants who regularly engage in rapid weight loss practices, as they represent the target population for understanding the effects of RWL.

Exclusion Criteria:

- Athletes with known musculoskeletal disorders or chronic pain conditions, as these could confound the Pressure Pain Threshold (PPT) results.
- Use of pain medication, performance-enhancing drugs, or other pharmaceutical aids during the study period, as these could influence pain perception and resilience levels.

### 2.2 The psychological resilience scale

The psychological Resilience Scale for Adults, developed by Friborg et al. (2005), and conducted Turkish validity and reliability by Basim and Cetin (2011) was used as the data collection tool. In the scale, ‘structural style’ (3,9,15,21) and ‘future perception’ (2,8,14,20) have 4 items each; ‘family harmony’ (5,11,17,23,26,32), ‘self-perception’ (1,7,13,19,28,31,) and ‘social competence’ (4,10,16,22, 25,29) are measured with 6 items each and ‘social resources’ (6,12,18,24,27,30,33) is measured with 7 items.

### 2.3 Measurement of pressure pain threshold

**Figure 1:**
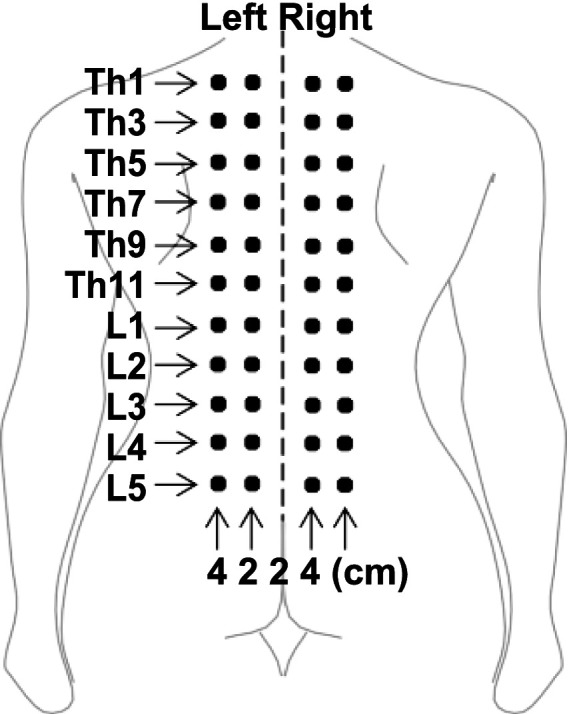
The measurement points. Bilateral paraspinal muscles of the thoracolumbar area (2 and 4 cm apart from midline at spinal segments Th1, Th3, Th5, Th7, Th9, Th11, L1, L2, L3, L4, and L5, i.e., 4 points × 11 segments = 44 points) were randomly tested to cancel order effect (Hanada et al., 2022).

Participants were positioned prone on a bed for the measurements. Pressure pain threshold (PPT), defined as the lowest pressure that induces pain, was assessed using a commercially available pressure algometer with a flat circular rubber tip of 1 cm^2^ surface area (JTECH Commander) (Fischer, 1986; Reeves et al., 1986; Fischer, 1987; Hanada et al., 2022). The measurements targeted bilateral paraspinal muscles in the thoracolumbar region at specific spinal segments (Th1, Th3, Th5, Th7, Th9, Th11, L1, L2, L3, L4, and L5) at distances of 2 cm and 4 cm from the midline (Figure 1). A total of 44 points (4 points × 11 segments) were marked with a pen, and a single point was randomly tested once using a random number generator to minimize measurement order effects (Balaguier et al., 2016). Pressure was applied perpendicular to the muscle surface at a rate of approximately 98 kPa/s (Farasyn and Meeusen, 2005), until the participants reported experiencing pain. The perpendicular application reduced the edge effect of the rubber tip on the algometer. To prevent temporal summation, measurements were spaced about 20 s apart (Nie et al., 2009). Each session lasted around 15 min per participant on an experimental day. The maximum pressure threshold, defined by the algometer, was 980 kPa, but all measured PPT values were below this limit.

The study was conducted over a one-month period preceding a major competition. All participants were assessed at two time points: (1) Before Rapid Weight Loss (BRWL), approximately one month before the competition, and (2) After Rapid Weight Loss (ARWL), on the morning of the official weigh-in. During the BRWL phase, baseline body mass, psychological resilience, and pressure pain threshold (PPT) measurements were recorded. Participants then underwent their usual training and RWL protocols, which included dietary restrictions, dehydration strategies, and increased physical activity. On the competition day, following the RWL phase, post-test measurements of body mass, psychological resilience, and PPT values were taken under identical conditions to minimize variability. The data collection process was standardized, with all participants evaluated in the same order using calibrated equipment to ensure reliability. The psychological resilience scale was administered via structured interviews, while PPT measurements targeted thoracolumbar paraspinal regions using a validated algometer. This procedure ensured consistency and reliability in assessing the physiological and psychological impacts of RWL (Table 1).

**Table 1:** Data collection process.

1st measurement Before rapid weight loss (BRWL)	2nd measurement After rapid weight loss (ARWL)
1 month before the competition	Competition day (Weight-in morning)
Taking body measurements, pressure pain threshold measurement, application of the psychological resilience scale	

### 2.4 Statistical analysis

Before starting the analysis, the assumption of normality was tested. According to the results of the Kolmogorov–Smirnov test (*p* > 0.05), the data followed a normal distribution. A Paired Samples t-test was conducted to compare the pre-test and post-test parameters. All statistical calculations were performed using the SPSS 26 ® software package. The magnitude of the changes observed in the study was classified according to Cohen’s criteria (Cohen, 1988; Cohen, 1992). Standardized mean difference and effect sizes (Cohen’s d) were categorized as follows: Trivial: < 0.2; Small: 0.2–0.3; Moderate: 0.4–0.8; Large: > 0.8. In the study the qualitative probabilistic terms was assigned using the following scale: <0.5%, most unlikely or almost certainly not; 0.5–5%, very unlikely; 5–25%, unlikely or probably not; 25–75%, possibly; 75–95%, likely or probably; 95–99.5%, very likely; >99.5%, most likely or almost certainly (Hopkins, 2007).

## 3 Results

The results presented in Table 2 show that significant increases were observed in the subdimensions of structured style (*t* = −2.543, *p* = 0.015), social competence (*t* = −2.910, *p* = 0.006), future orientation (*t* = −2.619, *p* = 0.011), and social resources (*t* = −2.560, *p* = 0.013) after RWL. However, no significant changes were found in the dimensions of family cohesion (*t* = −0.825, *p* = 0.415) and personal strength (*t* = −1.796, *p* = 0.081). These findings are partially in favor of the study’s aims and hypotheses, supporting the expectation that certain resilience subdimensions would improve during the RWL process due to structured routines and perceived social support, while others, such as family cohesion and personal strength, might remain stable.

**Table 2:** The comparison of psychological resilience before and after RWL in kickboxers (*n* = 39).

Subdimensions	Mean ± sd	*t*	*p*	Cohen’s *d*	Effect size	Change (%)
Structured style (pre)	3.82 ± 0.50	−2.543	0.015	0.36	Small	4.71
Structured style (post)	4.00 ± 0.50					
Social competence (pre)	3.90 ± 0.56	−2.910	0.006	0.41	Moderate	5.64
Social competence (post)	4.12 ± 0.53					
Family cohesion (pre)	3.82 ± 0.83	−0.825	0.415	0.18	Small	3.66
Family cohesion (post)	3.96 ± 0.61					
Personal strength (pre)	4.00 ± 0.56	−1.796	0.081	0.28	Small	4.0
Personal strength (post)	4.16 ± 0.58					
Future orientation (pre)	3.78 ± 0.68	−2.619	0.011	0.34	Small	5.82
Future orientation (post)	4.00 ± 0.62					
Social resources (pre)	3.62 ± 0.77	−2.560	0.013	0.47	Moderate	9.39
Social resources (post)	3.96 ± 0.60					

In Table 3, unranked athletes demonstrated significant improvements in structured style (*t* = −3.259, *p* = 0.005, d = 0.84, Change = 10.54%), social competence (*t* = −3.662, *p* = 0.002, *d* = 0.61, Change = 6.33%), future orientation (*t* = −2.542, *p* = 0.022, *d* = 0.64, Change = 10.05%), and social resources (*t* = −2.971, *p* = 0.009, *d* = 0.96, Change = 14.99%) after RWL. These findings align with the hypothesis that RWL would enhance resilience dimensions due to the structured and demanding nature of training routines. Conversely, ranked athletes did not show significant changes in these dimensions, suggesting that prior competitive success might buffer the psychological effects of RWL. For ranked athletes, changes in structured style (*t* = −0.105, *p* = 0.917, Change = 0.27%) and social competence (*t* = −1.533, *p* = 0.140, Change = 4.81%) were minor and not statistically significant. Similarly, no significant changes were observed in family cohesion or personal strength for either group, indicating that these dimensions might be less responsive to short-term interventions like RWL. These results partially support the study’s hypotheses. While unranked athletes benefited more from RWL in terms of resilience development, the lack of significant changes in certain dimensions and among ranked athletes highlights the variability in psychological responses to RWL.

**Table 3:** The comparison of psychological resilience based on the success in the previous tournament.

	Subdimensions	Mean ± sd	*t*	*p*	Cohen’s *d*	Effect size	Change (%)
For athletes who placed in the rankings (n = 23)	Structured style (pre)	3.76 ± 0.44	−0.105	0.917	0.02	Small	0.27
	Structured style (post)	3.77 ± 0.49					
	Social competence (pre)	3.74 ± 0.60	−1.533	0.140	0.31	Small	4.81
	Social competence (post)	3.92 ± 0.58					
	Family cohesion (pre)	3.60 ± 0.81	−1.031	0.314	0.33	Small	6.39
	Family cohesion (post)	3.83 ± 0.57					
	Personal strength (pre)	3.92 ± 0.61	−0.761	0.455	0.12	Small	2.04
	Personal strength (post)	4.00 ± 0.69					
	Future orientation (pre)	3.71 ± 0.70	−1.132	0.271	0.10	Small	1.89
	Future orientation (post)	3.78 ± 0.62					
	Social resources (pre)	3.43 ± 0.78	−0.925	0.365	0.23	Small	4.37
	Social resources (post)	3.58 ± 0.46					
For athletes who did not place in the rankings (*n* = 18)	Structured style (pre)	3.89 ± 0.58	−3.259	0.005	0.84	Large	10.54
	Structured style (post)	4.30 ± 0.33					
	Social competence (pre)	4.11 ± 0.45	−3.662	0.002	0.61	Moderate	6.33
	Social competence (post)	4.37 ± 0.33					
	Family cohesion (pre)	4.10 ± 0.78	−0.075	0.941	0.03	Small	0.49
	Family cohesion (post)	4.12 ± 0.65					
	Personal strength (pre)	4.12 ± 0.47	−1.78	0.094	0.58	Moderate	5.83
	Personal strength (post)	4.36 ± 0.32					
	Future orientation (pre)	3.88 ± 0.66	−2.542	0.022	0.64	Moderate	10.05
	Future orientation (post)	4.27 ± 0.51					
	Social resources (pre)	3.87 ± 0.69	−2.971	0.009	0.96	Large	14.99
	Social resources (post)	4.45 ± 0.36					

According to the results presented in Table 4, a significant decrease in Pressure Pain Threshold (PPT) values was observed across all thoracolumbar segments after weight loss (*p* < 0.001). Cohen’s d values indicate that these changes had a large effect size (*d* = 2.10–2.36). The largest PPT decrease was recorded in the L5 segment (*t* = −10.45; *d* = 2.36; Change = −11.04%), reflecting the pronounced sensitivity of this region. In contrast, the smallest decrease was observed in the Th1 segment (*t* = −8.23; d = 2.10; Change = −13.28%), although this still represents a substantial reduction. The findings support the study’s hypothesis that RWL would increase mechanical pain sensitivity due to biomechanical loading and dehydration. The consistent reductions across all segments indicate the pervasive impact of RWL on thoracolumbar regions. The gradient of change from Th1 to L5 may be attributed to regional anatomical differences, with lumbar muscles experiencing higher mechanical stress during physical activity. These results align with the study’s aim of evaluating the effects of RWL on pain sensitivity and reinforce the utility of PPT measurements in identifying biomechanical and neurophysiological alterations associated with weight loss.

**Table 4:** Segmental PPT values measured at 2 cm distance before and after weight loss (*n* = 39).

Segment	2 cm before Mean ± SD	2 cm after Mean ± SD	*t*	*p*	Cohen’s d	Effect size	Change (%)
Th1	472.63 ± 26.38	409.86 ± 31.85	−8.23	0.001	2.10	Large	−13.28
Th3	483.24 ± 26.61	420.12 ± 32.68	−8.45	0.001	2.12		−13.06
Th5	493.97 ± 27.43	431.09 ± 33.52	−8.67	0.001	2.15		−12.73
Th7	504.23 ± 27.77	441.34 ± 34.12	−8.89	0.001	2.18		−12.47
Th9	514.63 ± 28.48	451.62 ± 34.97	−9.12	0.001	2.20		−12.24
Th11	525.47 ± 29.12	462.23 ± 35.67	−9.34	0.001	2.23		−12.03
L1	536.26 ± 29.53	472.85 ± 36.12	−9.56	0.001	2.25		−11.82
L2	547.11 ± 30.17	483.54 ± 36.76	−9.78	0.001	2.28		−11.62
L3	558.25 ± 30.57	494.32 ± 37.23	−10.01	0.001	2.31		−11.45
L4	569.13 ± 31.12	505.12 ± 37.89	−10.23	0.001	2.34		−11.25
L5	579.86 ± 31.63	515.87 ± 38.45	−10.45	0.001	2.36		−11.04

According to the results presented in Table 5, Pressure Pain Threshold (PPT) values measured at a distance of 4 cm from the spine midline significantly decreased in all segments after weight loss (*p* < 0.001). Cohen’s d values indicate that these changes had a large effect size (*d* = 2.05–2.30). The largest decrease in PPT values was observed in the L5 segment (*t* = −9.78; *d* = 2.30; Change = −12.15%), while the smallest decrease was recorded in the Th1 segment (*t* = −7.45; *d* = 2.05; Change = −13.28%). These findings are consistent with the study’s hypothesis, confirming that RWL increases mechanical pain sensitivity across thoracolumbar segments. The uniform decrease in PPT values reflects the widespread impact of weight loss-induced biomechanical and physiological changes. The gradient of change, with a greater decrease in lower lumbar segments compared to thoracic segments, can be attributed to higher mechanical loading and localized stress experienced by lumbar muscles. This supports the study’s aim of evaluating the regional differences in pain sensitivity during the RWL process, emphasizing the importance of PPT as a reliable tool for assessing musculoskeletal changes.

**Table 5:** Segmental PPT values measured at 4 cm distance before and after weight loss (*n* = 39).

Segment	4 cm before Mean ± SD	4 cm after Mean ± SD	*t*	*p*	Cohen’s *d*	Effect size	Change (%)
Th1	427.61 ± 23.86	370.82 ± 28.82	−7.45	0.001	2.05	Large	−13.28
Th3	437.22 ± 24.07	380.10 ± 29.56	−7.78	0.001	2.08		−13.06
Th5	446.93 ± 24.82	389.98 ± 30.88	−8.01	0.001	2.10		−12.74
Th7	457.21 ± 25.13	399.23 ± 31.12	−8.22	0.001	2.14		−12.68
Th9	467.61 ± 25.76	408.45 ± 31.83	−8.45	0.001	2.16		−12.65
Th11	478.31 ± 26.19	418.11 ± 32.47	−8.67	0.001	2.18		−12.59
L1	489.09 ± 26.57	427.98 ± 32.91	−8.89	0.001	2.21		−12.49
L2	499.90 ± 27.13	437.86 ± 33.56	−9.12	0.001	2.23		−12.41
L3	511.13 ± 27.65	448.02 ± 34.12	−9.34	0.001	2.25		−12.35
L4	521.89 ± 28.17	457.56 ± 34.67	−9.56	0.001	2.28		−12.33
L5	532.68 ± 28.61	467.98 ± 35.12	−9.78	0.001	2.30		−12.15

## 4 Discussion

This study is one of the few to examine the effects of RWL on the levels of psychological resilience and mechanical pain sensitivity of elite kickboxers. The findings indicate that RWL increases pain sensitivity in the thoracolumbar region and creates differences in psychological resilience subscales. These findings, in line with previous literature, support the extensive effects of rapid weight loss on both physiological and psychological processes. Our study makes an important contribution to understanding the levels of these effects and their long-term implications for athletes’ performance and health.

According to the study findings a significant decrease in pressure pain threshold (PPT) values was observed in all segments after weight loss (*p* < 0.001). Cohen’s d values indicate that these changes had a large effect size (*d* = 2.10–2.36) also the percentage change is between −13.28% and − 11.04%. The largest PPT decrease was recorded in the L5 segment, supported by an effect size of *d* = 2.36. The lowest decrease was found in the Th1 segment (*d* = 2.10) (Table 4), indicating that PPT values measured at a distance of 4 cm from the spine midline significantly decreased in all segments after weight loss (*p* < 0.001). Cohen’s d values reveal that these changes also had a large effect size (*d* = 2.05–2.30) also the percentage change is between −13.28% and − 12.15%. The largest change was observed in the L5 segment (*t* = −9.78, *d* = 2.30). The lowest change was recorded in the Th1 segment (*t* = −7.45, *d* = 2.05) (Table 5). These findings suggest that the process of weight loss increases mechanical pain sensitivity, especially in the thoracolumbar region. The highest decrease in the L5 segment and the lowest decrease in the Th1 segment may be due to regional anatomical differences and biomechanical loading levels. The erector spinae muscles in the lumbar region are under high mechanical stress, which may lead to a more pronounced tenderness in this region. These results highlight the substantial impact of RWL on the levels of mechanical pain sensitivity, particularly in the lumbar region, likely due to biomechanical loading and hydration deficits. In Leźnicka et al. (2016), combat sports athletes showed significantly higher pain tolerance in CPT (Cold Pressor Test) and PPT tests (*p* < 0.001). In PPT, the pain threshold of the athletes was found to be higher; however, no significant difference was found in CPT. 91.43% of combat athletes were able to withstand >10 kg/cm^2^ of pressure, compared to 57.46% of non-athletes. During the CPT, combat athletes endured for 99.5 s, whereas non-sport athletes endured for an average of 86.0 s. In another study, the probability of high pain threshold was significantly higher in combat athletes (26%) than in non-athletes (1%) (p < 0.001). Combat athletes have higher pain tolerance due to their regular physical activity and intense training (Leźnicka et al., 2023). In another study similar to the present study, PPT values were higher in the lumbar region (e.g., L3-L5) compared to the thoracic region (e.g., Th1-Th3) in healthy and physically active young men. PPT values were higher in muscles closer to the midline of the spine (2 cm) than in muscles further away (4 cm). Length-increasing contractions caused increased muscle tenderness and mechanical hyperalgesia was more pronounced in the lower thoracic and lumbar regions. This study also suggests that PPT maps can be used in the management of exercise-induced muscle pain and can be a guide in rehabilitation processes (Hanada et al., 2022). Hanada et al. (2022) and the present study show similar findings. In this study, it was concluded that the compressive pain threshold in the lumbar and thoracic region decreased in elite kickboxers during the rapid weight loss period due to the intensive training period and rapid weight loss 1 month before the competition (Tables 4, 5). Anatomically, the trapezius muscle, which forms the most superficial layer of back skin, is distributed over a large cervicothoracic area, while the thoracolumbar fascia (TLF) and the aponeurosis of the latissimus dorsi muscle are known to cover the superficial lumbar area, which contains dense collagen fibers (Willard et al., 2012). Muscle-tendon regions or areas with collagen fibers are less sensitive to pressure than muscle belly regions (Nie et al., 2005; Andersen et al., 2006). This explains the higher PPT values in the lumbar region. The distribution and sensitivity of nociceptive afferents in tissues in the thoracolumbar region can be used as a neurophysiological basis for determining PPT values. In humans and rodents, the TLF in the lumbar region is densely innervated by nociceptive afferent fibers (Bove & Light, 1995; Tesarz et al., 2011; Barry et al., 2015; Mense, 2019). In mice, spinal dorsal horn neurons receive inputs from the TLF (Taguchi et al., 2008; Hoheisel et al., 2011). In human participants, injection of hypertonic saline into the TLF caused greater and longer-lasting pain sensation compared to injection into subcutaneous tissue or muscle (Schilder et al., 2014).

The levels of psychological resilience were also examined through changes in subdimensions. The findings show significant increases in the subdimensions of “structured style,” “social competence,” “future orientation,” and “social resources” after RWL (*p* < 0.05). On the other hand, no significant change was observed in “family cohesion” and “personal strength” subdimensions. These results are consistent with similar studies in the literature and reveal that athletes exhibit different psychological responses during the RWL process (Table 2).

The positive effects of RWL on “structured style” and “social competence” may be associated with athletes’ tendency to develop an organized approach to stressful situations. Sarkar and Fletcher (2014) highlighted the positive effects of resilience on stress management and performance and stated that a structured style supported by organizational skills plays an important role in competitive success. Furthermore, increases in the dimension of “social competence” suggest that the support athletes perceive from their social environment may be strengthened during the RWL period. Freeman and Rees (2009) showed that social support increases psychological resilience in athletes and improves their capacity to cope with stress.

The increases observed in the “future orientation” subdimension indicate that athletes develop a positive outlook toward the future, which increases their psychological resilience. Sarkar and Fletcher (2014) showed that having positive expectations for the future enables athletes to cope with stress and optimize their performance. This aligns with the study’s hypothesis that future orientation would improve during the RWL process, driven by goal-oriented behaviors and optimism. In this context, maintaining positive expectations during the RWL process may increase psychological resilience.Increases in the “social resources” dimension indicate that athletes perceive more support from their social environment. Coffee and Rees (2011) emphasized the positive effects of social resources on emotional regulation and coping strategies in athletes. The increases in social resources observed in this study suggest that athletes actively use social support and that this support increases their psychological resilience. However, changes in the dimensions of “family cohesion” and “personal strength” were not significant, suggesting that these dimensions may be less sensitive to changes in athletes’ resilience levels. As described by Meggs et al. (2019), personal characteristics may affect athletes’ resilience, but the fact that these dimensions did not change visibly during the RWL process may be due to the more fixed nature of individual characteristics.

Resilience refers to an individual’s capacity to maintain internal balance in the face of traumatic events or stressful situations, and it is often defined as the “ability to recover” from stress, trauma, or deprivation (Sisto et al., 2019). In sports, resilience is a critical attribute that enables athletes to adapt to and overcome various stressors, which can be categorized as competitive, organizational, or personal in nature. For instance, during high-stakes competitions like the Olympics, resilience has been linked to key factors such as positive personality traits, motivation, self-confidence, focus, and perceived social support (Sarkar and Fletcher, 2014).

In this study, subdimensions such as future perception, social resources, and structural style suggest that athletes who achieved rankings in previous tournaments displayed higher psychological resilience (Table 3). This could be attributed to the confidence developed through their success. Confidence, a core component of resilience, plays a pivotal role in managing stress and pressure, particularly in competitive sports (Gucciardi et al., 2011). Moreover, it has been highlighted as a crucial element in the resilience-stress-performance framework observed in elite athletes, including Olympic champions (Sarkar and Fletcher, 2014). Kickboxers who achieved prior success might have leveraged this confidence to build greater psychological resilience during subsequent competitive periods (Morris and Bone, 2024).

Personality traits further contribute to resilience. Defined as “relatively enduring patterns of thoughts, feelings, and behaviors,” personality traits significantly impact cognitive processes and athletic performance (Gould and Maynard, 2009; Roberts, 2009). Positive personality traits, such as conscientiousness and emotional stability, have been shown to foster resilience by enhancing an athlete’s ability to focus, regulate emotions, and cope with challenges. These traits may explain how successful kickboxers were able to maintain higher resilience levels. Optimism also plays a significant role in fostering resilience. Massey et al. (2011) described optimism as a persistent expectation of favorable outcomes, while (Buchanan and Seligman, 1995) defined it as an explanatory style for interpreting events. Optimistic athletes tend to exhibit lower levels of pre-competition anxiety (Wilson et al., 2002), better emotional regulation during competitions (Gaudreau and Blondin, 2004), and task-focused coping mechanisms after performance declines (Grove and Heard, 1997). This aligns with the findings of Coffee and Rees (2011), who reported that optimistic athletes recover more effectively from failures. In this study, the observed link between optimism and future perception suggests that successful athletes maintain an optimistic outlook, which reinforces their psychological resilience.

The aims of this study, which included examining the levels of resilience and pain thresholds, were partially supported by these findings. Specifically, it was hypothesized that PPT values would decrease significantly, and subdimensions of resilience associated with structured routines and social support would improve. However, the one-month follow-up period during the RWL process may have been too short to fully observe the persistence of these changes, particularly in the dimensions of “family cohesion” and “personal strength.” Future studies with extended follow-up durations are necessary to verify these observations.

In summary, the findings of this study support the hypothesis that RWL has multidimensional effects, including significant decreases in PPT values and varied improvements in resilience subdimensions. The observed changes contribute to a deeper understanding of the interplay between physiological and psychological processes during RWL. However, the study acknowledges the limitation of a short follow-up period, which may have affected the full exploration of resilience responses. Future research should aim to address this limitation by extending the follow-up duration and exploring individualized intervention strategies to optimize both physical and mental well-being during RWL.

## 5 Limitations and strengths

The limitation of this study is that it focuses solely on the kickboxing discipline. Future studies should include athletes from different sports disciplines to gain a broader understanding of the effects of the RWL period across various contexts. Another limitation is the short duration of the follow-up during the RWL period, defined in this study as one month. While this duration was sufficient to capture significant changes in both psychological resilience and mechanical pain sensitivity, previous research suggests that extended follow-up periods, such as 8–12 weeks, may provide more comprehensive insights into the long-term physiological and psychological impacts of RWL (Drid et al., 2024; Lakicevic et al., 2020). For instance, Lakicevic et al. (2020) highlighted that prolonged monitoring can help identify recovery patterns post-RWL, which are crucial for designing optimal athlete management strategies. Future research should consider extending the monitoring period to encompass both the RWL phase and subsequent recovery periods, offering a holistic perspective on the effects of rapid weight loss.

## Data Availability

The raw data supporting the conclusions of this article will be made available by the authors, without undue reservation.
